# Supplementary material for: Impact of Blueberry Consumption on the Human Fecal Bileacidome: A Pilot Study of Bile Acid Modulation by Freeze-Dried Blueberry
Source: Nutrients. 2022 Sep 17;14(18):3857. doi: 10.3390/nu14183857 (PMC9501813; doi:10.3390/nu14183857)
Supplement: Supplementary file 1 [file nutrients-14-03857-s001.zip › nutrients-1884171-supplementary.pdf]

IMPACT OF BLUEBERRY CONSUMPTION ON THE HUMAN FECAL BILEACIDOME: A PILOT STUDY

William Gagnon, Véronique Garneau, Jocelyn Trottier, Mélanie Verreault, Charles Couillard, Denis Roy, André Marette, Jean-Philippe Drouin-Chartier, Marie-Claude Vohl and Olivier Barbier

Supplemental table S1: Comparison of the baseline BA profile in fecal samples from male and female volunteers.

Bile acids	Men (n= 11)		Women (n=13)		Mean Diff.	Ajusted p value
	Mean	SEM	Mean	SEM		
CA	0,1051	± 0,0585	0,2595	± 0,1255	0,1544	>0,999
CDCA	0,0595	± 0,0267	0,1282	± 0,0453	0,0687	>0,999
DCA	2,6994	± 0,5642	3,0877	± 0,3504	0,3883	>0,999
LCA	2,3580	± 0,4183	2,6709	± 0,2440	0,3128	>0,999
HDCA	0,0093	± 0,0018	0,0083	± 0,0015	-0,0010	>0,999
HCA	0,0016	± 0,0008	0,0029	± 0,0011	0,0014	>0,999
UDCA	0,0221	± 0,0093	0,0410	± 0,0152	0,0189	>0,999
GCA	0,0244	± 0,0162	0,0087	± 0,0030	-0,0156	>0,999
GCDCA	0,0141	± 0,0068	0,0070	± 0,0013	-0,0070	>0,999
GDCA	0,0153	± 0,0064	0,0114	± 0,0024	-0,0039	>0,999
GLCA	0,0003	± 0,0001	0,0003	± 0,0001	0,0000	>0,999
GUDCA	0,0014	± 0,0005	0,0006	± 0,0002	-0,0008	>0,999
TCA	0,0090	± 0,0051	0,0206	± 0,0145	0,0116	>0,999
TCDCA	0,0052	± 0,0024	0,0092	± 0,0052	0,0040	>0,999
TDCA	0,0124	± 0,0062	0,0460	± 0,0204	0,0336	>0,999
TLCA	0,0002	± 0,0001	0,0026	± 0,0012	0,0025	>0,999
TUDCA	0,0002	± 0,0001	0,0010	± 0,0007	0,0008	>0,999
TOTAL BA	5,4549	± 1,0199	6,4739	± 0,6204	1,0190	>0,999
Unconjugated	5,2549	± 0,9853	6,1985	± 0,6111	0,9436	>0,999
Taurine-conjugated	0,0270	± 0,0132	0,0794	± 0,0402	0,0524	>0,999
Glycine-conjugated	0,0555	± 0,0296	0,0281	± 0,0062	-0,0274	>0,999
Primary	0,2172	± 0,0901	0,4333	± 0,1827	0,2160	>0,999
Secondary	5,0857	± 0,9654	5,8189	± 0,5461	0,7333	>0,999
6α-hydroxylated	0,0108	± 0,0018	0,0112	± 0,0023	0,0004	>0,999
Total CA	0,1385	± 0,0668	0,2889	± 0,1406	0,1504	>0,999
Total CDCA	0,0789	± 0,0289	0,1444	± 0,0491	0,0655	>0,999
Total DCA	2,7273	± 0,5700	3,1452	± 0,3601	0,4179	>0,999
Total LC	2,3587	± 0,4185	2,6739	± 0,2444	0,3152	>0,999
Total HDCA	0,0093	± 0,0018	0,0083	± 0,0015	-0,0010	>0,999
Total HCA	0,0016	± 0,0009	0,0029	± 0,0011	0,0013	>0,999

Values are presented as mean concentration (nmol/mg of feces) of the 24 pre- and post-diet samples ± SEM (standard error of the mean).

Mean Diff., namely the difference between pre- vs post-treatment were calculated for each of the participants (11 men and 13 women), and values represent the mean±SEM.

P values were calculated using Mann-Whitney test than adjusted for multiple comparisons using the Bonferroni-Dunn method. Bile acids composition analysis were performed as detailed in the materials and method section.

CA: cholic acid; CDCA: chenodeoxycholic acid; LCA: lithocholic acid; DCA: deoxycholic acid; HDCA: hyodeoxycholic acid; HCA: hyocholic acid; UDCA: Ursodeoxycholic acid. G: glyco; T: tauro.

Supplemental table S2: Comparison of the bile acid profile in fecal samples from male and female volunteers harvested after the 8-week freeze-dried blueberry consumption period.

Bile acids	Men (n= 11)		Women (n=13)		Mean Diff.	Ajusted p value
	Mean	SEM	Mean	SEM		
CA	0,5167	± 0,3585	0,0671	± 0,0242	-0,4496	>0.999
CDCA	0,2443	± 0,1419	0,0325	± 0,0102	-0,2118	>0.999
DCA	1,8429	± 0,3261	2,3693	± 0,3279	0,5265	>0.999
LCA	1,7667	± 0,2542	2,1686	± 0,1894	0,4019	>0.999
HDCA	0,0056	± 0,0008	0,0072	± 0,0013	0,0016	>0.999
HCA	0,0024	± 0,0011	0,0014	± 0,0005	-0,0009	>0.999
UDCA	0,0529	± 0,0264	0,0508	± 0,0286	-0,0021	>0.999
GCA	0,0391	± 0,0192	0,0184	± 0,0034	-0,0207	>0.999
GCDCA	0,0222	± 0,0090	0,0127	± 0,0023	-0,0095	>0.999
GDCA	0,0142	± 0,0020	0,0147	± 0,0029	0,0006	>0.999
GLCA	0,0003	± 0,0000	0,0003	± 0,0001	0,0000	>0.999
GUDCA	0,0021	± 0,0006	0,0014	± 0,0005	-0,0007	>0.999
TCA	0,0359	± 0,0210	0,0087	± 0,0019	-0,0273	>0.999
TCDCA	0,0350	± 0,0202	0,0051	± 0,0011	-0,0299	>0.999
TDCA	0,0191	± 0,0115	0,0214	± 0,0074	0,0023	>0.999
TLCA	0,0006	± 0,0003	0,0015	± 0,0007	0,0009	>0.999
TUDCA	0,0023	± 0,0016	0,0006	± 0,0004	-0,0017	>0.999
TOTAL BA	4,7414	± 0,8047	4,9157	± 0,5387	0,1744	>0.999
Unconjugated	4,4314	± 0,8005	4,6969	± 0,5316	0,2655	>0.999
Taurine-conjugated	0,0930	± 0,0483	0,0374	± 0,0109	-0,0556	>0.999
Glycine-conjugated	0,0778	± 0,0291	0,0475	± 0,0084	-0,0303	>0.999
Primary	0,8932	± 0,5258	0,1445	± 0,0403	-0,7487	>0.999
Secondary	3,6436	± 0,5431	4,5758	± 0,4952	0,9322	>0.999
6α-hydroxylated	0,0080	± 0,0011	0,0086	± 0,0013	0,0006	>0.999
Total CA [9]	0,5918	± 0,3696	0,0942	± 0,0281	-0,4977	>0.999
Total CDCA [9]	0,3024	± 0,1591	0,0503	± 0,0125	-0,2520	>0.999
Total DCA [9]	1,8768	± 0,3312	2,4055	± 0,3322	0,5286	>0.999
Total LCA [9]	1,7678	± 0,2541	2,1704	± 0,1895	0,4026	>0.999
Total HDCA [9]	0,0056	± 0,0008	0,0072	± 0,0013	0,0015	>0.999
Total HCA [9]	0,0025	± 0,0012	0,0014	± 0,0005	-0,0011	>0.999

Values are presented as mean concentration (nmol/mg of feces) of the 24 pre- and post-diet samples ± SEM (standard error of the mean).

Mean Diff., namely the difference between pre- vs post-treatment were calculated for each of the participants (11 men and 13 women), and values represent the mean±SEM.

P values were calculated using Mann-Whitney test adjusted for multiple comparisons using the Bonferroni-Dunn method. Bile acids composition analysis were performed as detailed in the materials and method section.

CA: cholic acid; CDCA: chenodeoxycholic acid; LCA: lithocholic acid; DCA: deoxycholic acid; HDCA: hyodeoxycholic acid; HCA: hyocholic acid; UDCA: Ursodeoxycholic acid. G: glyco; T: tauro.
